# Editorial: Molecular Dynamics Simulations of Metalloproteins and Metalloenzymes

**DOI:** 10.3389/fchem.2021.789299

**Published:** 2021-11-29

**Authors:** Kshatresh Dutta Dubey, Binju Wang, Yubing Si, Syed Tarique Moin

**Affiliations:** ^1^ Department of Chemistry and Center for Informatics, School of Natural Sciences, Shiv Nadar University Delhi-NCR, Greater Noida, India; ^2^ State Key Laboratory of Physical Chemistry of Solid Surfaces and Fujian Provincial Key Laboratory of Theoretical and Computational Chemistry, College of Chemistry and Chemical Engineering, Xiamen University, Xiamen, China; ^3^ College of Chemistry, Zhengzhou University, Zhengzhou, China; ^4^ Third World Center for Science and Technology, H.E.J. Research Institute of Chemistry, International Center for Chemical and Biological Sciences, University of Karachi, Karachi, Pakistan

**Keywords:** metalloenzyemes, MD simulation, metal ions, conformational dynamics and allostery, QM/MD simulation, metalloproteins

## Introduction of the Topic

Metalloenzymes are a superfamily of enzymes that possess metal ions as a cofactor and catalyze a plethora of reactions such as oxidations, reductions, biosynthesis, and drug metabolism. These reactions are regulated by synchronized choreography of some strategic protein residues that act as a signal to communicate with the metal center to perform a specific function for the catalysis. Such choreography of the protein’s residues is, nowadays, routinely studied by Molecular Dynamics (MD) Simulations. This special issue covers two important aspects of simulations of metalloenzymes: 1) accurate and efficient modeling of metal ions during simulations and 2) a few applications of molecular dynamics simulations to study the conformational changes *en route* to catalysis by metalloenzymes/metalloproteins. These articles are written by some leading researchers in this field. Here, we briefly introduce these articles in this new collection.

## Models in Metalloenzymes

### A New Algorithm for Fast and Accurate Potentials for Zinc Containing Proteins

The development of a potential energy function that provides accuracy at QM standard and efficiency at MM scale is a long-time goal for computational chemists. In this special issue, Zhang and coworkers have developed an algorithm based on an enhanced self-organizing incremental high dimensional neural network—deep potential model for zinc-containing metalloproteins (Xu et al.). The authors have shown that calculated atomic forces, potential energy, and atomic charges are in good agreement with quantum mechanical calculations. Therefore, the proposed model of the Zhang group could have a significant impact on the simulations of the metalloenzymes since it incorporates the polarization effect in the simulation with the speed at the MM level.

### A New Fluctuating 12-6-4 Ion Model for Metal Ions

An accurate modeling of metal ions is crucial during MD simulations. The previously proposed 12–6 Lennard-Jones non-bonded ion model by the Merz’s group was efficient but couldn’t produce the experimental hydration-free energy which was further corrected by the 12-6-4 model by the same group. However, it was found that the upgraded 12-6-4 model overestimates the coordination number of highly charged metal ions. In the current issue, Pengfei Li proposed a fluctuating charge 12-6-4 model which further rectifies such overestimations of coordination numbers (Li). In fact, the author shows that the MD simulations using fluctuating charge 12-6-4 model successfully reproduced the results of the previous *ab-initio* MD simulations.

## Applications of MD Simulations in Functions of Metalloenzymes

### MD Reveals Promiscuity in CYP76AH1

Cytochrome P450 is nature’s most versatile metalloenzyme, and they show the widest substrate specificity among all enzymes known so far. An article by Wu and coworkers inside this special issue beautifully explains such versatility in CYP76H1 using a multiscale simulation approach supplemented by Density Functional Theory (DFT) calculations (Qiu et al.). The study provides key insights about the conformational changes that cause substrate promiscuity due to D301E and V479F mutations in the native enzyme.

### MD Simulations Help to Identify Metabolites in Human Indoleamine Dioxygenase

2,3-hIDO is a heme-containing dioxygenase that inserts dioxygen to tryptophan and cleaves the pyrrole ring for carcinogenic effect in the human body. In a study inside the issue, Sen and coworkers synthesized few novel inhibitors that block this carcinogenic effect which was further validated by an *in-vitro* study (Chauhan et al.). They implemented MD simulations and QM/MM calculations to predict the mechanism of inhibition. The study beautifully shows how theoretical calculations can complement experimental drug discovery projects.

### Simulation Uncovers the Picture of CO_2_ Hydration in Carbonic Anhydrase

In this article of the current issue, Wang and Cao’s group used MD simulations and QM/MM calculations to reveal the complete enzymatic mechanism of CO_2_ hydration by Carbonic Anhydrase (Fu et al.). The study highlights a key role of His64 residue which regulates the enzymatic activity by toggling between inward and outward orientations. The study provides excellent state of the art of classical MD and quantum MD simulations.

### Simulations Reveal a Key Structural Motif in the Mechanism of NagB

NagB (glucosamine-6-phosphate deaminase) catalyzes the catabolism of amino sugars in biological systems. In an article on this issue, Wang and Zhao’s group used extensive MD simulations supplemented by enhanced sampling methods like umbrella sampling to study the role of active site residues which play a key role in the function of the NagB enzyme (Zhang et al.). Using classical MD simulations, they identified a key lid motif that plays a pivotal role by maintaining an open conformation that enables easy access of the substrate to the active site.

### A Dynamical Insight of the Metal Binding in Cu/Zn Superoxide Mutase

The bindings of Cu and Zn Ions are vital in pathogenesis of many neurodegenerative disease. Using classical MD simulations for superoxide dismutase (SOD1) that causes Amyotrophic lateral sclerosis (ALS), Timucin et al. shows how enhanced flexibility due to H63R and K136A could weaken the metal binding and hence causes toxicity in SOD1 proteins (Timucin et al.).

